# Failure of Passive Transfer in Camel Calves: 4 Cases (2010-2019)

**DOI:** 10.1155/2022/8182648

**Published:** 2022-04-23

**Authors:** Amanda James, Joe Smith, Julie Sheldon, Ricardo Videla

**Affiliations:** ^1^College of Veterinary Medicine, University of Tennessee, Knoxville, TN, USA; ^2^Large Animal Clinical Sciences, College of Veterinary Medicine, University of Tennessee, Knoxville, TN, USA; ^3^Biomedical Sciences, College of Veterinary Medicine, Iowa State University, Ames, IA, USA; ^4^Small Animal Clinical Sciences, College of Veterinary Medicine, University of Tennessee, Knoxville, TN, USA

## Abstract

Failure of passive transfer is a management concern for all ruminant species, but is not well described in the literature for camel calves. This case series presents four camel calves (Camelus dromedarius and Camelus bactrianus) referred to a North American veterinary teaching hospital for diagnosis and management of failure of passive transfer. Diagnostics utilized included hematology, serum biochemistry, and immunologic methods as described for crias. Management included antimicrobial, anti-inflammatory, and plasma transfusion therapies. Three of the four calves survived to discharge, and common diagnostic practices such as evaluation of total solids, total protein, immunoglobulin G, and sodium sulfite appear to be correlate to passive transfer status in these four calves. Xenotransfusion with llama plasma was well tolerated by two calves, and xenotransfusion with bovine plasma was well tolerated by an additional calf in this study. An additional work is necessary to develop validated breakpoints for diagnosis of passive transfer status in camel calves.

## 1. Introduction

Failure of passive transfer (FPT), also known as failure of passive immunity, is a common health concern in neonatal ruminants that results from inadequate colostrum absorption. Treatment, diagnoses, and outcomes of FPT have been reported for lambs [1], kids [2], calves [3, 4], and crias [5, 6]; however, veterinary literature is lacking in cases of FPT in neonatal Old World camel species. Old World camel species include the dromedary (*Camelus dromedarius*) and the Bactrian (*Camelus bactrianus*), which are native to the Middle East region and Northern Africa, and central Asia, respectively. However, the population of camels elsewhere, such as Australia [5] and North America [6], is increasing. Despite the large numbers of camels worldwide, there is currently a paucity of cases reported in the literature describing the management of failure of passive transfer in neonatal camels.

Failure of passive transfer is the failure of young animals to obtain an adequate amount of colostral immunoglobulins after parturition. Ruminants have epitheliochorial placentas, which prevents the uptake of immunoglobulins prior to birth [7]. Uptake occurs through the ingestion of colostrum and absorption in the small intestines. One study found that at the time of parturition, the IgG concentration of camelid colostrum is high at 58.6 g/l. This concentration rapidly decreases to 38.8 g/dl at 24 hours postparturition and 16.5 g/dl 72 hours postparturition [8]. It is imperative that camelid crias are suckling adequately within the first 24-hour period when the concentration is high, as failure of passive transfer is linked to a higher risk of developing infectious disease and fatality [7, 9, 10].

To the authors' knowledge, this is the first reported case series of failure of passive transfer (FPT) in camel calves seen in a North American Veterinary teaching hospital. The following four cases, referred to the University of Tennessee's Veterinary Medical Center (UTVMC) between the years of 2010 and 2019, represent FPT diagnosis, outcome, and management in camel calves.

## 2. Case Presentation

A retrospective search was done through UTVMC record database for camel cases that were diagnosed as failure of passive transfer. Search functions included a designation of camel as species, as well as the selection of age for neonates. Records were then checked for the diagnosis of FPT and then further evaluated. Cases that lacked a clear diagnosis of FPT were excluded.

### 2.1. Case 1

A 1-day-old, 25 kg, male dromedary calf was presented for weakness and lateral recumbency. The calf experienced dystocia during his birth the night prior; the calf was not interested in nursing and got progressively weaker.

On initial examination, the calf was laterally recumbent with a corneal ulcer in the left eye. Pulse was 110 beats per minute (normal range) and respiratory rate 30 breaths per minute (normal range). Rectal temperature was 37.3°C (normal range), blood glucose was low at 29 mg/dl (reference range: 86-140 [11]), and plasma total solids was 3.6 mg/dl. Based on this plasma total solids value, which was low compared to values reported for crias (>5.0 g/dl being suggestive of adequate passive transfer [4]), as well as historical dystocia and lack of observed nursing, failure of passive transfer was diagnosed.

Therapy was initiated with a continuous rate infusion of balanced polyionic (Norm-R) IV fluids and 2% dextrose at a rate of 60 ml/kg/day (64 ml/hr). Ceftiofur sodium (Naxcel^TM^) was administered IV every 12 hours at a dosage of 4.4 mg/kg. Three units of plasma (provided by client, unknown source) were transfused. Triple antibiotic ointment was applied to the left eye every 6 hours.

On day two of hospitalization, the calf's blood glucose was within normal values (111 mg/dl); the calf started ambulating and was observed to have dropped fetlocks in both pelvic limbs. On day 3, the calf's posttransfusion IgG level was reported to be 800 mg/dl by radial immunodiffusion, suggesting FPT as the plasma transfusion did not elevate the total concentration to values associated with adequate passive transfer in other ruminant species. At this point, the calf was discharged from the hospital.

### 2.2. Case 2

A three-day-old, 36 kg, female, dromedary camel was presented for regurgitation, lethargy, and eventual collapse. After parturition, the calf was rejected by her dam and was administered bovine colostrum. The calf was also administered milk replacer and fluids—the volume, type, and route of each are unknown. After two days of on-farm supplementation and supportive care, the client presented the calf to the teaching hospital.

On presentation, the calf was unresponsive and laterally recumbent. Rectal temperature was 38.8°C, heart rate was 123 beats per minute, and respiratory rate was 32 breaths per minute. Mucous membranes were hyperemic, and capillary refill time was two seconds. Packed cell volume (PCV) was 28%, and total solids measured 5.0 g/dl (Table 1). On initial bloodwork, she was hypernatremic (165 mEq/l), hyperglycemic (520 mg/dl), and neutropenic (360/*μ*l) with a plasma protein of 4.1 g/dl. She was diagnosed with presumptive neonatal sepsis and failure of passive transfer. Blood was collected for blood culture and susceptibility testing.

The calf was given ceftiofur sodium (Naxcel™) intravenously at 5 mg/kg every 12 hours. The calf was started on intravenous fluids, 0.45% saline with 2.5% dextrose at a rate of 83 ml/kg/day (125 ml/hr). Additionally, 1.1 mg/kg of flunixin meglumine was administered intravenously every 12 hours. Regular insulin was later added to her fluids at a rate of 0.025 U/kg/hr for control of hyperglycemia at the initiation of fluid therapy.

The calf was found nonresponsive later that same day and was bleeding from her trachea and did not respond to cardiopulmonary resuscitation. Postmortem examination revealed moderate diffuse hepatic lipidosis, moderate multifocal acute hepatic necrosis with intralesional bacilli, severe multifocal acute pulmonary necrosis with intralesional bacilli, and mild acute anterior chamber hemorrhage. *Bacillus* septicemia was diagnosed based on samples collected for culture at necropsy, as well as the blood culture results.

### 2.3. Case 3

A one-day-old, female, 50 kg, Bactrian camel presented for weakness and suspected FPT. The calf was through a dystocia; during delivery, the dam sustained a vaginal tear. Following the birth, the dam was reluctant to allow nursing. The calf was fed a unit of powdered colostrum replacer on the first day, and the following day was fed 1-1.5 pints of sheep milk replacer every three hours. The calf became progressively weaker and was assessed by the primary veterinarian on. Total solids were 5 g/dl, and complete blood count was within normal limits. The calf was treated with an injection of ceftiofur crystalline-free acid (Excede™). The calf's condition continued to deteriorate, and she was referred to the teaching hospital.

On presentation, she was bradycardic (42 beats per minute) and her respiratory rate was 30 breaths per minute. PCV was 23%, total solids 5.2 g/dl, and urine specific gravity 1.042. A sodium sulfite test was performed with a score of 2+ (Table 1). Hypopyon and anterior uveitis were observed in the left eye. The calf was diagnosed with FPT and endophthalmitis uveitis. The calf was started on lactated ringer's intravenous fluids at a rate of 40 ml/kg/day (83 ml/hr). She was given 1 ml of iron dextran intramuscularly once. Vitamin B12 (1 ml) was injected subcutaneously every 12 hours.

On the third day of hospitalization, the calf was observed to have extensor tendon laxity bilaterally in the thoracic limbs so splinting was employed to provide support. Complete blood count revealed toxic changes. IgG level was measured via radial immunodiffusion (RID) at <200 mg/dl. A transfusion of two liters of llama plasma was initiated. The treatment protocol at this time included the following: ceftiofur (Naxcel™) 4.4 mg/kg IV every 12 hours, flunixin meglumine 0.5 mg/kg IV every 12 hours, atropine ophthalmic and neo-poly-bac ointments in her left eye every 6 hours, amikacin 10 mg/kg IV every 24 hours with a 250 ml fluid bolus, and sucralfate 20 mg/kg orally every 8 hours. On the fifth day of hospitalization, her blood culture demonstrated no bacterial growth at three days. On the sixth day of hospitalization, recheck RID level results were received and were 300 mg/dl. This value was confirmed with protein electrophoresis. She was administered 2 additional liters of llama plasma. An ophthalmology consultation confirmed hypopyon and anterior uveitis with flare in her left eye. At this point, the triple antibiotic ointment was changed to a neomycin-polymyxin B-dexamethasone ointment. On the eighth day of hospitalization, the calf's RID measured 550 mg/dl. The left eye had improved with less tearing and flare observed. A hemogram performed at this time yielded no evidence of toxic changes. The calf was discharged on day 9 after initial presentation. Follow-up at 10 days after discharge indicated a healthy appearing calf with a good appetite. Long-term follow-up with the client indicated that the calf was healthy one year after discharge when it was sold.

### 2.4. Case 4

A 1-day-old, 35 kg, male, dromedary camel was presented after being rejected by its dam and refusing a bottle. The calf was born one day prior and received colostrum from the dam as well as by tube feeding from the owner. At the time of presentation, the calf had not yet been observed to urinate.

On presentation, physical exam revealed rectal temperature of 38.1°C, heart rate of 144 beats per minute, and respiratory rate of 54 breaths per minute, Borborygmi were decreased; mucous membranes were pink and tacky, with a capillary refill time of two seconds noted. The calf had bilateral carpal valgus and tendon laxity in all four fetlock joints, which was noted to be more severe in the forelimbs. The umbilicus appeared normal. Ultrasound revealed comet tail lesions in all lung fields, and prescapular lymph nodes were enlarged. Complete blood count revealed leukopenia, and serum biochemistry revealed a presumptive hypernatremia (154 mmol/l). Presumptive diagnoses were sepsis and aspiration pneumonia.

The calf was administered 2 units of llama plasma. Afterwards, 0.45% saline was intravenously administered with 2.5% dextrose at a rate of 82 ml/kg/day (120 ml/hr). Ceftiofur sodium (Naxcel™) was administered at a dosage of 5.5 mg/kg IV every 12 hours.

Initial urinalysis showed trace bilirubin, trace ketones, occult blood (3+), and protein. Blood glucose was consistently elevated so a continuous rate infusion of insulin (humulin) was started at 1 U/hr as previously reported for crias [12]. A sodium sulfite test was performed with a result of 2+ (Table 1). An RID test was submitted on day 4 and measured 540 mg/dl. Based on these values, the calf received a second llama plasma transfusion (2 units). At this time, the RID measured 1190 mg/dl. The calf had showed clinical improvement while in hospital and was discharged six days after initial presentation. The client indicated that approximately one year after discharge the calf was sold and had no apparent health issues until the time of sale.

## 3. Discussion

While there are multiple references for failure of passive transfer in sheep, goats, cattle, llamas, and alpacas, to the authors' knowledge, there are no case series for failure of passive transfer described in camels. As previously mentioned, failure of passive transfer puts newborns at a higher risk for developing disease and a higher rate of mortality [7, 9, 10]. Comorbidities associated with the camelids in this study included sepsis, optic lesions, and pneumonia. This finding is consistent with a study following septic New World camelid crias, of which a large portion had septicemia [14].

Comorbidities associated with failure of passive transfer are not specific to camelids. Bovine calves diagnosed with failure of passive transfer have been found to be at a higher risk for developing bovine respiratory disease and bacterial pneumonia associated with *Pasteurella multocida* [15]. Lambs with failure of passive transfer are more likely to develop enterotoxemia associated with *Clostridium perfringens* types C and D [16]. Similarly, foals who do not receive adequate colostral immunoglobulins are more likely to develop septicemia, septic arthritis, and pneumonia [17]. Pneumonia and enteritis are also common comorbidities found in crias that have failure of passive transfer. More severe lesions that have been documented in crias include bacterial meningoencephalitis and brain abscessation due to *Escherichia coli* [18]. Additional physical examination findings in these four calves that coincide with other ruminant cases of failure of passive transfer include ocular abnormalities such as anterior chamber abnormalities (cases 2 and 3).

Case 1 was suspected to have failure of passive transfer through two methods: total solid measurement below 4.5 g/dl and an IgG measurement less than 100 mg/dl. Case 2 was evaluated using total solids and falls into the range where passive transfer status is not clear; however, the clinical presentation and history suggest this calf's IgG passive transfer status was suboptimal (or insufficient). Case 3 was evaluated with three methods: total solids, IgG, and sodium sulfite. For case 3, measurements for IgG are repeatedly markedly low and consistent with failure of passive transfer; however, the total solid value is not definitive and the sodium sulfite value of 2+ would indicate IgG concentrations between 500 and 1500 mg/dl in other species. In case 4, the IgG measurement is initially inadequate but improves to successful transfer status. Similar to case 3, this sodium sulfite test could be consistent with adequate transfer status in bovine calves.

There are multiple tests available to determine an animal's IgG passive transfer status. An automated analyzer is used to determine serum total protein, with a value of 5.0 g/dl or greater found to be consistent with adequate passive transfer. Total solids levels, measured by refractometer, between 4.5 and 5.5 g/dl can be indicative of either failure of passive transfer or successful passive transfer, respectively, and should be interpreted cautiously. Measurements outside of this range are consistent with failure of passive transfer and successful passive transfer, respectively. Serum IgG concentration is performed via radial immunodiffusion assays with a concentration of 1000 mg/dl or greater considered adequate in crias [9]. Sodium sulfite compares turbidity when serum is added to varying concentrations (14%, 16%, and 18%) of sodium sulfite and is graded 0 (no turbidity), 1 (turbidity in 18%), 2 (turbidity in 16% and 14%), and 3 (turbidity in 18%, 16% and 14%). A value of 2+ can be consistent with IgG > 1000 mg/dl and adequate transfer [13] but can include a range of 500-1500, and as such could also indicate failure of passive immunity. A serum chemistry analyzer is used to measure GGT; however, its values do not always directly correlate to the serum level of IgG and clinicians should use caution when using GGT concentrations as a sole predictor of adequate passive transfer [19, 20].

An interesting therapy was transfusing of llama plasma in cases one and three as well as the use of bovine colostrum in the second case. Transfusions of bovine plasma and administration of bovine colostrum have been used for the management of FPT in giraffe calves [21]. Xenotransfusion of whole bovine blood has been used in an emergency transfusion in a wildebeest calf as well as experimentally investigated as a potential treatment in goats [22, 23] suggesting that bovine plasma could be used in those species as well. Due to the challenges in finding a camel plasma donor, clinicians should consider the use of plasma from other closely related species when camel plasma is unavailable.

Limitations of this study include the small sample size and its retrospective nature. An additional limitation is the different method of confirmation of FPT utilized for every case. While this study used comparative values from similar species (South American Camelids), it should be noted that failure of passive transfer of immunity is not well researched in Old World camelids, and future research should focus on immunoglobulin and other values of camel calves deemed to have received an appropriate amount of adequate colostrum. Clinicians should recognize the importance of clinical signs and history of potential FPT in cases where validated diagnostic results are not currently known.

In conclusion, accurate recognition of and testing for failure of passive transfer in neonatal camel calves is vital due to the high risk for developing infectious diseases and death. Further research is needed to determine the best course of therapy and which species of ruminant can provide the most successful plasma replacement.

## Figures and Tables

**Table 1:** Results of various diagnostics for the four camel calves with comparisons to values for crias and calves [9, 13].

Method	Species reference	Reference value	Case #1	Case #2	Case #3	Case #4
Total protein	Crias (llama and alpaca)	FPT ≤ 5.0 g/dl	3.6 g/dl	4.1 g/dl	NA	NA
Total solids	Crias (llama and alpaca)	FPT ≤ 4.5 g/dl; adequate ≥ 5.5 g/dl	NA	5.0 g/dl	5.2 g/dl	NA
IGG (RID)	Crias (llama and alpaca)	FPT ≤ 1,000 mg/dl	800 mg/dl (post-transfusion)	NA	300 mg/dl, then 550 mg/dl (posttransfusion)	540 mg/dl, then 1190 mg/dl (posttransfusion)
Sodium sulfite	Calves	1+ (FPT) to 3+ (indicating successful transfer)	NA	NA	2+	2+

FPT: failure of passive transfer; IGG: immunoglobulin G; RID: radial immunodiffusion.

## Data Availability

All data is present in the study.

## References

[1] Sawyer M., Willadsen C., Osburn B., McGuire T. (1977). Passive transfer of colostral immunoglobulins from ewe to lamb and its influence on neonatal lamb mortality. *Journal of the American Veterinary Medical Association*.

[2] O'brien J., Sherman D. (1993). Serum immunoglobulin concentrations of newborn goat kids and subsequent kid survival through weaning. *Small Ruminant Research*.

[3] Tibary A., Fite C., Anouassi A., Sghiri A. (2006). Infectious causes of reproductive loss in camelids. *Theriogenology*.

[4] Whitehead C. E. (2009). Management of neonatal llamas and alpacas. *Veterinary Clinics: Food Animal Practice.*.

[5] Murray M. D., Snowdon W. A. (1976). The role of wild animals in the spread of exotic diseases in Australia. *Australian Veterinary Journal*.

[6] Locklear T. R., Videla R., Breuer R. (2021). Presentation, clinical pathology abnormalities, and identification of gastrointestinal parasites in camels (Camelus bactrianus and Camelus dromedarius) presenting to Two North American Veterinary Teaching Hospitals. A retrospective study: 1980–2020. *Science*.

[7] Bishr A. M., Magdub A. B., Abuzweda A.-B. R. (2013). The appropriate time required for newborn calf camel to get optimal amount of colostrums immunoglobulin (IgG) with relation to levels of cortisol and thyroxin. *International Journal of Animal and Veterinary Sciences.*.

[8] Kamber R., Farah Z., Rüsch P., Hässig M. (2000). The supply of newborn camel foals (Camelus dromedarius) with immunoglobulin G. *Archiv für Thierheilkunde*.

[9] Weaver D. M., Tyler J. W., Marion R. S., Wallace L. M., Nagy J. K., Holle J. M. (2000). Evaluation of assays for determination of passive transfer status in neonatal llamas and alpacas. *Journal of the American Veterinary Medical Association*.

[10] Salhi I., Bessalah S., Mbarek S. B. (2015). Passive transfer of maternal immunity in the dromedary (Camelus dromedarius), involvement of heavy chain antibodies. *Tropical Animal Health and Production*.

[11] Islam S., Ferdous J., Rahman M. K., Akter S., Hassan M. M., Islam A. (2019). Reference values for hematological and serum biochemical parameters of dromedary camel (Camelus dromedarius) in sub-tropical climate of Bangladesh. *Advances in Animal and Veterinary Sciences.*.

[12] Buchheit T. M., Sommardahl C. S., Frank N., Roberson J. R. (2010). Use of a constant rate infusion of insulin for the treatment of hyperglycemic, hypernatremic, hyperosmolar syndrome in an alpaca cria. *Journal of the American Veterinary Medical Association*.

[13] Tyler J. W., Hancock D. D., Parish S. M. (1996). Evaluation of 3 assays for failure of passive transfer in calves. *Journal of Veterinary Internal Medicine*.

[14] Dolente B. A., Lindborg S., Palmer J. E., Wilkins P. A. (2007). Culture-positive sepsis in neonatal camelids: 21 cases. *Journal of Veterinary Internal Medicine*.

[15] Peek S. F., Ollivett T. L., Divers T. J. (2018). *Respiratory diseases*.

[16] Simpson K. M., Callan R. J., Van Metre D. C. (2018). Clostridial abomasitis and enteritis in ruminants. *Veterinary Clinics: Food Animal Practice.*.

[17] Raidal S. L. (1996). The incidence and consequences of failure of passive transfer of immunity on a thoroughbred breeding farm. *Australian Veterinary Journal*.

[18] Tsur I., Harmelin A., Dvir I., Yanai J. (1996). Meningoencephalitis and brain abscessation due to Escherichia coli in a 2 week old alpaca cria. *Australian Veterinary Journal*.

[19] Johnson A. L., Probst C. W., Decamp C. E. (2001). Comparison of trochlear block recession and trochlear wedge recession for canine patellar luxation using a cadaver model. *Veterinary Surgery*.

[20] Johnston N., Parish S., Tyler J., Tillman C. (1997). Evaluation of serum gamma-glutamyltransferase activity as a predictor of passive transfer status in crias. *Journal of the American Veterinary Medical Association*.

[21] Dixon C. E., Bedenice D., Restifo M., Mazan M., Brewer P., Knafo S. E. (2021). Neonatal intensive care of 10 hospitalized giraffe calves (*Giraffa camelopardalis*) requiring hand-rearing. *Journal of Zoo and Wildlife Medicine*.

[22] Buck R. K., Stegmann G. F., Poore L. A., Shaik T., Gray T., Zeiler G. E. (2018). Xenotransfusion with packed bovine red blood cells to a wildebeest calf (*Connochaetes taurinus*). *Journal of the South African Veterinary Association*.

[23] Smith J. S., Viall A. K., Breuer R. M. (2021). Preliminary investigation of bovine whole blood xenotransfusion as a therapeutic modality for the treatment of anemia in goats. *Frontiers in Veterinary Science.*.

